# Endoscopic endonasal resection of neurohypophyseal granular cell tumor: a case report and review of 88 published cases

**DOI:** 10.3389/fendo.2026.1796860

**Published:** 2026-04-15

**Authors:** Yahui Zhang, Ming-chen Xie, Hao Han, Xun Xie, Wenyue Zhang, Jianhua Cheng, Jian Xu

**Affiliations:** Department of Neurosurgery, The Affiliated Hospital of Qingdao University, Qingdao, China

**Keywords:** endoscopic transnasal transsphenoidal surgery, granular cell tumor, neurohypophysis, pituitary stalk, posterior pituitary, sellar region

## Abstract

**Background:**

Granular cell tumor (GCT) of the neurohypophysis is a rare tumor originating from the posterior pituitary/infundibulum, classified as WHO grade I. Due to its imaging characteristics being highly similar to those of common sellar lesions, diagnosis typically relies on histopathology and immunohistochemistry.

**Case presentation:**

A 43-year-old male presented with a one-month history of pulsatile temporal headaches without significant visual complaints. Endocrine tests showed reduced levels of growth hormone and prolactin. CT scan of the head and MRI of the sellar region revealed a well-defined solid mass in the sellar/suprasellar region (approximately 20×19×21 mm), compressing the optic chiasm and closely associated with the pituitary stalk. The patient underwent endoscopic transnasal transsphenoidal tumor resection. Intraoperatively, the tumor was soft but highly vascular, and adhered to the pituitary stalk, requiring meticulous hemostasis and en bloc removal. Postoperative MRI confirmed total resection. Histologically, the tumor consisted of polygonal to spindle-shaped cells with abundant eosinophilic granules in the cytoplasm. Immunohistochemistry showed positivity for TTF-1, S100, and CD68, with a Ki-67 index of approximately 5%, while SOX10 was negative, supporting the diagnosis of neurohypophyseal GCT.

**Literature review:**

We reviewed 88 published case reports to compare demographic characteristics, clinical presentations, endocrine abnormalities, treatment strategies, and recurrence rates.

**Conclusion:**

Neurohypophyseal GCT should be included in the differential diagnosis of solid sellar/suprasellar masses associated with the pituitary stalk. Endoscopic transnasal transsphenoidal resection is effective, but the rich vascular supply and adhesion to the pituitary stalk can increase the surgical difficulty. Due to the potential for late recurrence, long-term follow-up is recommended.

## Highlights

- Solid sellar/suprasellar masses closely related to the pituitary stalk should include neurohypophyseal GCT in the differential diagnosis.
- Diagnosis relies on histology and immunohistochemistry (TTF-1 and CD68 positivity, along with prominent granular cytoplasm, is particularly indicative).
- Endoscopic transnasal transsphenoidal resection is effective, but the tumor’s rich blood supply and adhesion to the pituitary stalk can increase surgical difficulty.
- Even with a low Ki-67 index, recurrence may occur, and long-term follow-up is recommended.

## Introduction

Granular cell tumor (GCT) of the neurohypophysis is a rare lesion originating from the posterior pituitary and/or infundibulum. Although “granular cell tumors” can occur at various anatomical sites, sellar neurohypophyseal GCTs exhibit relatively distinct biological and immunophenotypic characteristics. The 2021 WHO classification of central nervous system tumors grouped it with other TTF-1 positive neurohypophyseal tumors (such as pituicytoma and spindle cell oncocytoma) within the same spectrum (1). When the tumor causes a space-occupying effect, compressing the optic pathways or affecting hypothalamic-pituitary function, it can lead to significant symptoms and complications.

Preoperative diagnosis is often challenging. Sellar/suprasellar GCTs can mimic pituitary adenomas, craniopharyngiomas, meningiomas, germ cell tumors, and inflammatory lesions on imaging. Therefore, diagnosis typically relies on intraoperative or postoperative histological identification of “granular cytoplasm” features, combined with immunophenotypic classification (2). From a surgical perspective, these tumors often have a rich blood supply and are tightly adherent to the pituitary stalk, presenting unique challenges for complete resection (3).

This article reports a case of a middle-aged male with a neurohypophyseal GCT treated with endoscopic transnasal transsphenoidal resection. We summarize 88 previously published case reports, outlining key diagnostic and therapeutic points, discussing the controversies surrounding adjuvant radiotherapy, and emphasizing the importance of long-term follow-up.

## Case report

### Patient information

A 43-year-old male with no significant history of chronic diseases presented in March 2024 with a one-month history of pulsatile temporal headaches. He denied dizziness, nausea, vomiting, polyuria, polydipsia, or visual disturbances, and had no relevant family history.

### Clinical examination

The patient was alert and oriented, with normal cognitive function. Pupils were equal and reactive to light (approximately 3 mm), with normal direct and indirect light reflexes. Eye movements were intact, and no focal neurological deficits were observed.

### Diagnostic evaluation

**Figure 1:**
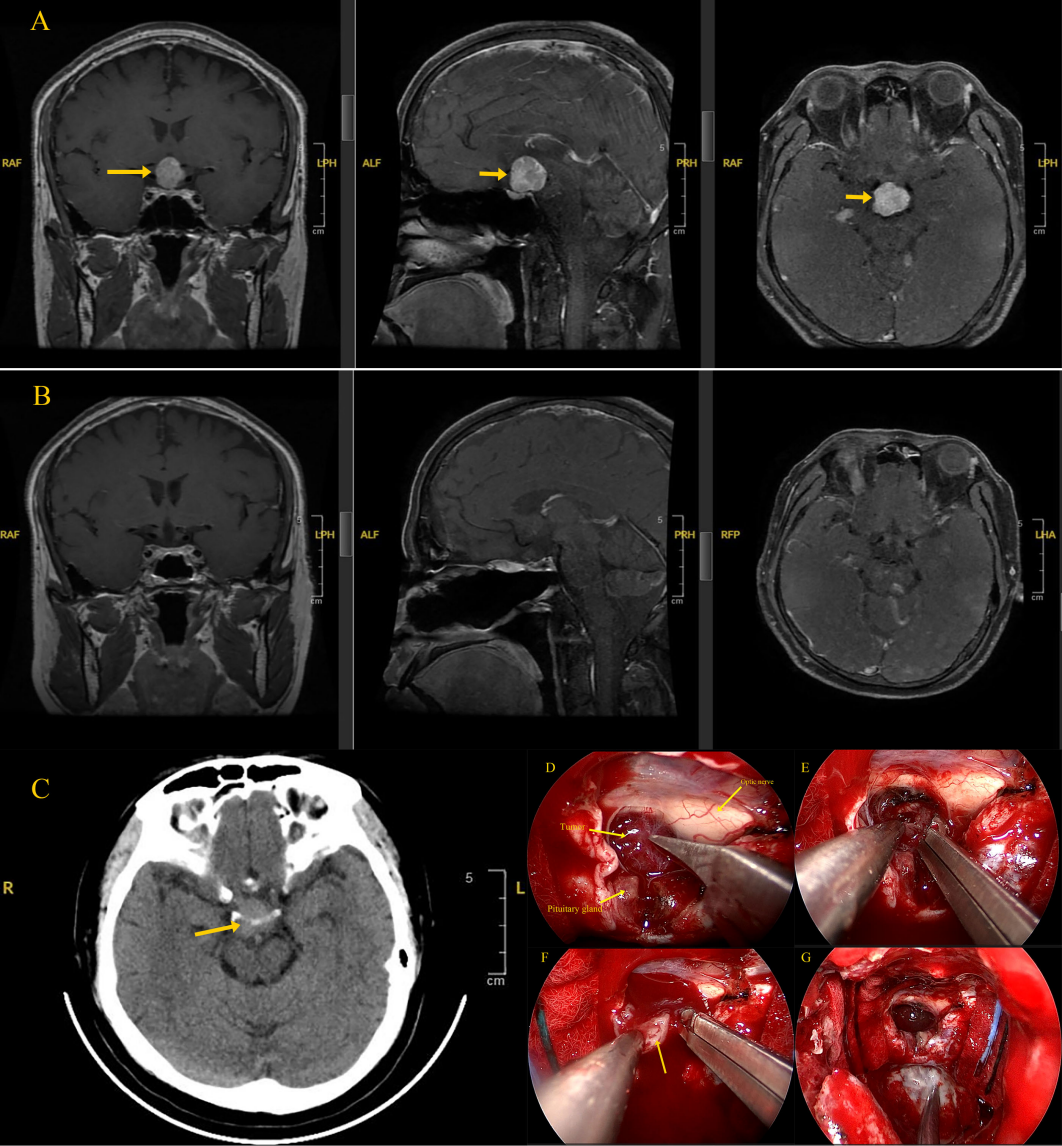
**(A)** preoperative sagittal, coronal, and vertical-axis MRI of the brain showing a tumor in the saddle region; **(B)** postoperative sagittal, coronal, and vertical-axis MRI of the brain showing tumor removal; **(C)** Cranial CT showing a space-occupying lesion in the saddle region; **(D)** The positional relationship between the tumor and surrounding tissues; **(E)** Intraoperative endoscopic view showing tumor exposure with sparse vascular distribution on its surface and clear boundaries with adjacent tissues; **(F)** Intraoperative endoscopic view demonstrating extremely rich tumor blood supply, accompanied by significant bleeding during resection; **(G)** Intraoperative endoscopic images showing complete tumor removal, with no residual tumor tissue observed in the surgical area.

Serum hormone testing revealed decreased growth hormone (<0.03 ng/mL; reference range 0.03–2.47 ng/mL) and decreased prolactin (62.92 mIU/L; reference range 72.66–407.4 mIU/L) (**Table 1**). CT of the head revealed a mass in the sellar region. MRI of the sellar region showed a well-defined, round, solid mass with high signal intensity on T1-weighted images, measuring approximately 20×19×21 mm (superior-inferior × lateral × anterior-posterior) (**Figure 1**). The optic chiasm was compressed and displaced upward, and the pituitary stalk was unclear, suggesting a close relationship with the stalk. Based on imaging findings, the initial diagnosis was a tumor in the pituitary region.

**Table 1:** Key endocrine and immunohistochemistry results.

Item	Result	Reference/notes
Growth hormone (GH)	<0.03 ng/mL	0.03–2.47 ng/mL
Prolactin (PRL)	62.92 mIU/L	72.66–407.4 mIU/L
TTF-1	Positive	IHC
S100	Positive	IHC
CD68	Positive	IHC
Ki-67	~5%	–
SOX10	Negative	IHC

### Treatment

The patient underwent endoscopic transnasal transsphenoidal tumor resection. Intraoperatively, the tumor was primarily suprasellar, solid, soft in texture, and had very rich blood supply. It was tightly adherent to the pituitary stalk. The tumor was removed in fragments with meticulous hemostasis. Estimated blood loss was around 500 mL, and 400 mL of plasma was transfused intraoperatively. Reconstruction of the skull base was performed using a pedicled mucosal flap with vascular supply, reinforced with an artificial dura mater patch.

### Follow-up and outcome

**Figure 2:**
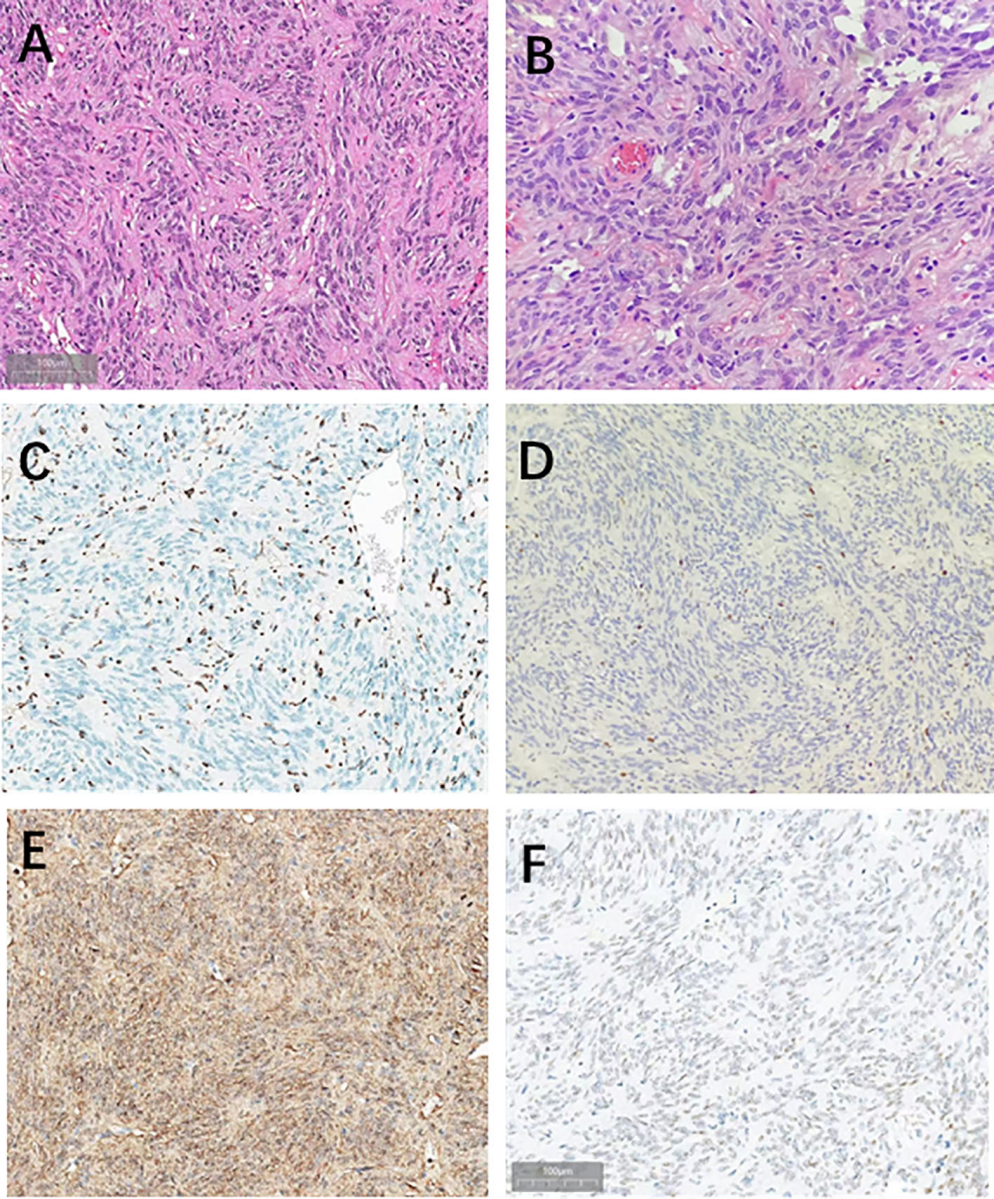
Hematoxylin and eosin stain of the tumor tissue in this case. Tumor cells are spindle-shaped, mildly anisotropic, with occasional nuclear schizophrenia. [**(A)** 20×; **(B)** 40×]; Immunohistochemistry results. **(C)** TTF-1 showed positive staining in the cytoplasm of tumor cells (20×); **(D)** Immunohistochemical results of s-100 were positive expressed in cells (20×); **(E)** CD68 is positive in tumor cells (20×); **(F)** Ki-67 showed positive staining in the cytoplasm of tumor cells. (About 5%,20×).

The patient recovered smoothly, with relief of the headache. Postoperative MRI confirmed complete tumor resection. Histology showed tumor cells with round to irregular nuclei, abundant eosinophilic granules in the cytoplasm, and mild atypia. Immunohistochemistry revealed positivity for TTF-1, S100, and CD68, with a Ki-67 index of approximately 5%, while SOX10 was negative, confirming the diagnosis of neurohypophyseal granular cell tumor (WHO grade I) (**Figure 2**). Long-term radiological and endocrine follow-up was recommended to monitor for recurrence and pituitary dysfunction. At the 2-year follow-up, the patient was doing well, with no evidence of recurrence. (**Table 2**).

**Table 2:** Timeline of the episode of care.

Date/period	Key events
Feb 2024	Onset of pulsatile temporal headache.
Mar 2024 (admission)	Neurologic exam largely normal; endocrine tests show low GH and low PRL.
Mar 2024 (workup)	CT/MRI reveal ~20×19×21 mm sellar/suprasellar mass compressing optic chiasm; stalk not clearly visualized.
Mar 2024 (surgery)	Endoscopic endonasal transsphenoidal resection; tumor highly vascular and adherent to stalk; EBL ~500 mL; reconstruction with pedicled flap.
Post-op	Headache resolved; MRI confirms gross-total resection; pathology/IHC consistent with neurohypophyseal GCT (WHO grade 1).
Planned	Long-term imaging and endocrine follow-up to monitor recurrence and pituitary dysfunction.
Follow-up	At the two-year follow-up, there was no recurrence.

### Perioperative key points

Given that neurohypophyseal GCTs often have a rich blood supply and are closely associated with the pituitary stalk, it is crucial to inform the patient about the potential risks of endocrine complications (such as diabetes insipidus and hypopituitarism) preoperatively. Due to adhesion to the pituitary stalk, gentle dissection of the tumor capsule is often limited. A “gradual decompression + strict hemostasis + repeated evaluation of the pituitary stalk-tumor interface” strategy is recommended to minimize the risk of hypothalamic-pituitary injury. Early dynamic monitoring of serum sodium and urine output, along with timely endocrine evaluation, is essential to identify transient or persistent pituitary dysfunction.

## Literature review

### Search strategy

We searched the PubMed database using combinations of the following terms (search period: January 1, 1981, to December 31, 2024): “neurohypophyseal granular cell tumor,” “sellar granular cell tumor,” “suprasellar granular cell tumor,” and related keywords. We also screened the reference lists of relevant articles. In addition, we searched a Chinese biomedical database (China National Knowledge Infrastructure [CNKI]; search period: January 1, 2016, to December 31, 2024). The inclusion criteria were: (1) published case reports/case series of sellar/suprasellar neurohypophyseal granular cell tumor; (2) histopathologically confirmed diagnosis; and (3) extractable data on demographic characteristics, clinical presentation, treatment, and follow-up. Exclusion criteria included non-neurohypophyseal granular cell tumors, reviews without individual patient data, and duplicate reports.

### Main findings

Among the 88 case reports (**Supplementary Table S1**), females slightly outnumbered males (57.3%). The peak age for diagnosis was between 40 and 60 years, with an overall average age of 49.25 ± 16.57 years (female: 51.15 ± 16.57; male: 46.57 ± 16.73). The most common initial symptoms were visual disturbances or visual field changes (53.4%) and headaches or dizziness (40.9%). Other symptoms included polyuria, menstrual changes, nausea, and vomiting. Reports on endocrine abnormalities varied. Among the 55 cases providing laboratory data, hyperprolactinemia (32.7%) was the most common, consistent with the “pituitary stalk effect.” Other abnormalities included decreased growth hormone, thyroid hormones, and adrenal axis hormones.

Immunohistochemical results showed significant variation across reports, possibly related to differences in the testing panels and historical classification changes. Among cases reporting this marker, 71.6% were positive for S100, 30.7% for CD68, and 27.3% for TTF-1. The TTF-1 positivity rate may be underestimated in earlier cases where it was not routinely tested. PAS positivity (41.0%) was also common. Nearly all cases underwent surgical treatment, with 10.2% receiving radiotherapy (either as primary or adjuvant treatment). In 56.8% of cases, there was documented compression or invasion of surrounding tissues. Among 44 cases with follow-up data, the recurrence rate was 4.5%.

### Other noteworthy observations

The tumor was most commonly described as located suprasellar or at the base of the pituitary stalk, with many cases involving both suprasellar and intrasellar regions; pure intrasellar tumors were rare. Cystic changes and calcification were reported infrequently, suggesting that “solid lesions” may be the typical imaging phenotype. Most researchers believe that symptoms and laboratory abnormalities are primarily due to local compression or involvement of the pituitary stalk, rather than hormone secretion by the tumor itself. Additionally, follow-up times varied considerably, with some reports noting delayed regrowth many years after initial surgery. This supports the recommendation for long-term follow-up even after complete resection with a low Ki-67 index.

## Discussion

Neurohypophyseal granular cell tumor (GCT) is a low-grade tumor originating from the posterior pituitary and/or infundibulum, and it accounts for a very small proportion of sellar tumors (approximately 0.5% based on published series) (1, 3). In clinical practice, the most common sellar/suprasellar masses are pituitary adenomas, craniopharyngiomas, and meningiomas, which means that neurohypophyseal GCT is often not the primary preoperative diagnosis (4). Its rarity, combined with the lack of specificity in imaging, means that most cases require postoperative histopathological confirmation (5). The present case, with a one-month history of pulsatile headache, did not have significant visual complaints; however, preoperative imaging revealed a well-defined sellar/suprasellar mass that compressed the optic chiasm and was closely associated with the pituitary stalk, accompanied by mild endocrine abnormalities. This fits the characteristics of neurohypophyseal tumors, which often present symptoms primarily due to space-occupying effects and hypothalamic-pituitary dysfunction.

In the review of 88 case reports, females were slightly more common (57.3%). The most common symptoms were vision/visual field changes (53.4%) and headaches/dizziness (40.9%). Among the cases with laboratory data, hyperprolactinemia was the most frequent finding, likely reflecting compression of the pituitary stalk (the “pituitary stalk effect”). Although hyperprolactinemia is more commonly reported in the literature on neurohypophyseal granular cell tumor (GCT)—typically attributed to pituitary stalk compression with reduced hypothalamic dopaminergic inhibition—our case presented with hypoprolactinemia, suggesting that the underlying pathophysiology may not be explained solely by the “pituitary stalk effect.” Instead, it may be more plausibly related to tumor-related compression or injury involving the normal anterior pituitary, the hypophyseal portal circulation, and the pituitary stalk–hypothalamic functional unit. This finding is consistent with the imaging studies and intraoperative findings in this case, namely that the tumor was closely associated with the pituitary stalk and exhibited significant adhesions. Notably, the concomitant decrease in growth hormone (GH) in this patient further supports impaired anterior pituitary reserve as a likely explanation. Because hypoprolactinemia is usually clinically subtle in males, its significance in this context lies primarily in indicating potential pituitary dysfunction, underscoring the need for long-term endocrine follow-up. The key takeaway is that when a solid sellar/suprasellar mass is associated with optic pathway compression, pituitary stalk involvement, and endocrine abnormalities, the differential diagnosis should not be limited to pituitary adenoma but should also include neurohypophyseal tumors and hypophysitis.

Imaging features of neurohypophyseal GCT typically show a solid, well-defined sellar/suprasellar mass that can grow upward and compress the optic chiasm (6). Its signal characteristics and enhancement patterns overlap significantly with pituitary adenomas, meningiomas, craniopharyngiomas, germ cell tumors, and inflammatory lesions. Some reports suggest CT hypodensity, T1 hyperintensity, and mild T2 hypointensity, but these features are not reliable enough to form preoperative diagnostic criteria (7–9). More practical clues include a tumor located in the posterior pituitary/infundibulum, closely related to the pituitary stalk, and predominantly solid in composition. However, a definitive diagnosis still requires histological confirmation.

Pathologically, GCT is characterized by sheets or nests of polygonal tumor cells with abundant eosinophilic granules in the cytoplasm, indicating a lysosome-rich phenotype (10, 11). Under the WHO framework, neurohypophyseal tumors (including pituicytoma, spindle cell oncocytoma, and GCT) generally express TTF-1, supporting a pituicyte lineage (12). However, these tumors overlap in morphology and some markers, so it is necessary to support the diagnosis of GCT with features such as “prominent granular cytoplasm + strong CD68 positivity.” S100 is frequently positive, and the absence of SOX10 can help exclude lesions of Schwann cell or melanocytic origin in specific contexts (10). Ki-67 is typically low, consistent with its low-grade biological behavior, but caution is needed in interpreting this, as tumors with low proliferative indices may still recur (5, 7).

Differential diagnosis: Pituitary adenoma, like neurohypophyseal granular cell tumor (GCT), can cause hypopituitarism and hyperprolactinemia due to the pituitary stalk effect. (13). On imaging, GCT typically arises in the suprasellar region involving the infundibulum,pituitary stalk and posterior pituitary and is often highly vascular, whereas pituitary adenomas are usually intrasellar, expand the sella, and may invade the cavernous sinus. Hypophysitis is also female-predominant (75.0%), with a higher female proportion than that reported in neurohypophyseal GCT. It shares overlapping features with GCT, including headache, hyperprolactinemia, and growth hormone deficiency (14). Preoperative imaging may help distinguish them: GCT often appears as an enhancing suprasellar mass (sometimes relatively hyperdense on CT), while hypophysitis more commonly shows symmetric enlargement of the pituitary gland and stalk with loss of the posterior pituitary T1 bright spot. Definitive diagnosis relies on postoperative histopathology and immunohistochemistry.

Regarding treatment, surgery remains the first-line option for neurohypophyseal GCTs. Endoscopic transnasal transsphenoidal approaches provide direct access to the sellar region, suprasellar cistern, and pituitary stalk area and have become the primary surgical method in many centers (15). However, neurohypophyseal GCTs are often technically challenging to remove. Previous reports have repeatedly emphasized the close adhesion to the pituitary stalk and hypothalamic structures, and they are often associated with significant vascularity. Both of these factors can limit the safety of aggressive dissection and increase the risk of complications (3). In our case, the tumor was soft but highly vascular and closely associated with the pituitary stalk, requiring fragment removal with meticulous hemostasis, highlighting the need for preoperative planning for blood loss and skull base reconstruction. Intraoperatively, dynamic assessment of the anatomical interface should guide the “maximum safe resection” boundary.

The extent of resection is closely related to prognosis. Multiple reports suggest that larger tumors or those undergoing subtotal resection are more likely to progress or recur, while total resection offers long-term control in a significant proportion of patients (16). However, the goal should be “maximum safe resection,” not unrelenting anatomical total resection, as excessive tension or damage to the pituitary stalk may lead to diabetes insipidus, hypopituitarism, or even hypothalamic injury (3, 16). Clinical decisions should be individualized, taking into account the degree of adhesion, bleeding tendency, and baseline endocrine status of the patient.

There is ongoing debate regarding adjuvant radiotherapy after subtotal resection. Some authors suggest radiotherapy for residual disease due to concerns about recurrence, while other reviews emphasize that the tumor is generally benign and lacks clear evidence of benefit from radiotherapy (17, 18). Furthermore, radiotherapy may result in delayed toxicity to the optic pathway and hypothalamic-pituitary axis. Due to the tumor’s rarity and varying indications, there is a lack of high-quality comparative studies. Therefore, the most reasonable approach is to assess on a case-by-case basis, considering the volume of residual disease, growth rate, proximity to the optic pathway, and patient factors (5). In our review, only a small proportion of cases (10.2%) reported receiving radiotherapy, indicating that it is not a routine strategy.

In practical decision-making after subtotal resection, whether the residual lesion grows and its distance from the optic nerve/optic chiasm is often crucial. If the residual lesion is stable on serial MRI and the patient is asymptomatic, observation with structured follow-up may be chosen. If growth or symptom recurrence is confirmed, reoperation or local radiotherapy may be considered. If radiotherapy is chosen, the potential for late adverse effects should be thoroughly discussed, and the treatment plan must strictly adhere to optic pathway dose limits. Based on these considerations, most authors prefer to reserve radiotherapy for recurrent or progressive residual tumors rather than routinely applying it to all patients with subtotal resection (5).

Regardless of the initial treatment, long-term follow-up is critical, as recurrence can occur, and may be late. A feasible follow-up strategy is to conduct early postoperative MRI to confirm the extent of resection, followed by regular (e.g., annual) imaging surveillance, with prompt evaluation for new headaches, visual symptoms, or endocrine changes. Given the tumor’s location and the risk of postoperative pituitary dysfunction, imaging follow-up should be combined with endocrine function monitoring.

### Limitations

This literature summary is based on published case reports, which are subject to reporting and publication biases. Diagnostic criteria and immunohistochemical panels have varied across different time periods, and reports on imaging sequences, endocrine tests, and follow-up durations are often incomplete or inconsistent. Therefore, the positive rates of various biomarkers, imaging patterns, and recurrence rates should be understood as descriptive conclusions rather than definitive estimates. Future establishment of multicenter prospective registries will help clarify the optimal surgical goals and the true value of radiotherapy for residual/recurrent lesions.

## Conclusion

Although rare, neurohypophyseal GCT should be considered when a solid sellar/suprasellar mass is located in the posterior pituitary-infandibulum region or closely related to the pituitary stalk. Imaging is crucial for evaluating the anatomical relationship but lacks specificity; definitive diagnosis requires histological and immunophenotypic confirmation. Endoscopic transnasal transsphenoidal surgery offers effective resection, but careful preoperative planning for vascularity and pituitary stalk adherence is necessary, with adherence to the principle of “maximum safe resection” and attention to hypothalamic-pituitary function preservation. Given the possibility of recurrence (including late recurrence), long-term imaging and endocrine follow-up are recommended, even after total resection.

## Patient perspective

After surgery, the patient reported complete relief of headache, returned to normal daily activities, and agreed to long−term follow−up. At the 2-year follow-up, the patient was doing well, with no evidence of recurrence.

## Data Availability

The original contributions presented in the study are included in the article/**Supplementary Material**, further inquiries can be directed to the corresponding author/s.
