# Supplementary material for: Endoscopic endonasal resection of neurohypophyseal granular cell tumor: a case report and review of 88 published cases
Source: Front Endocrinol (Lausanne). 2026 Apr 15;17:1796860. doi: 10.3389/fendo.2026.1796860 (PMC13124585; doi:10.3389/fendo.2026.1796860)
Supplement: Supplementary file 1 [file Table1.docx]

Table S1 Summary of previously reported cases of GCT in the saddle region

| **Author** | **Year** | **Age** | **Sex** | **Symptoms** | **Hormone** | **Treament** | **S-100** | **TTF-1** | **CD56** | **CD68** | **Ki-67(%)** | **Further positive IHC** | **Further negative IHC** | **Tumor Ivasion or Compression** | **Recurrence** |
| --- | --- | --- | --- | --- | --- | --- | --- | --- | --- | --- | --- | --- | --- | --- | --- |
| **Paulo R Benites Filho** | **2005** | **8** | **M** | **DV,HA,D** | **N** | **ST** | **-** | **ND** | **ND** | **ND** | **ND** | **PAS,NSE** | **Actin，GFAP** | **ND** | **ND** |
| **Filippo Gagliardi** | **2013** | **11** | **M** | **HA** | **IGF(L),FT3(L)** | **ST&MT** | **-** | **ND** | **ND** | **+** | **<1** | **PAS** | **GFAP,EMA,NSE,** | **Yes** | **ND** |
| **Yang Guoqiang** | **2011** | **14** | **M** | **DV,Polyuria** | **N** | **ST** | **+** | **ND** | **ND** | **+** | **ND** | **PAS,GFAP,** | **EMA** | **No** | **No** |
| **Ba Jianming** | **2007** | **14** | **M** | **Polyuria** | **N** | **ST&RT** | **+** | **ND** | **ND** | **ND** | **ND** | **PAS** |  | **Yes** | **No** |
| **Zhu Mingxin** | **2011** | **16** | **M** | **VFD** | **PRL(H)** | **ST** | **-** | **ND** | **ND** | **+** | **ND** | **GFAP,NSE** |  | **Yes** | **ND** |
| **S Ogata** | **2001** | **17** | **M** | **DV,VFD** | **PRL(H),LH(L)** | **ST** | **+** | **ND** | **ND** | **ND** | **10.1** | **P53,Vimentin** | **EMA,GFAP** | **Yes** | **ND** |
| **Vera Popovic** | **2007** | **21** | **F** | **HA,HM** | **PRL(H),IGF-1(L)GH(L)** | **ST** | **+** | **-** | **ND** | **ND** | **2.0** | **CD34,PAS** |  | **Yes** | **No** |
| **Mark D Wilkinson** | **2003** | **27** | **F** | **HM,VFD,L,Polyuria** | **ND** | **ST** | **-** | **ND** | **ND** | **ND** | **ND** |  | **GFAP,** | **Yes** | **No** |
| **Yi Zhang** | **2018** | **29** | **F** | **HM** | **PRL(H),E2(L)** | **ST** | **ND** | **ND** | **ND** | **ND** | **5.0** | **PAS** |  | **ND** | **No** |
| **Wim Maenhoudt** | **2020** | **29** | **M** | **ND** | **N** | **ST&MT** | **+** | **ND** | **ND** | **ND** | **ND** | **GFAP** |  | **Yes** | **No** |
| **Jia Si** | **2014** | **29** | **F** | **HM** | **ND** | **ST** | **+** | **ND** | **ND** | **-** | **ND** | **GFAP** | **NF** | **Yes** | **ND** |
| **Michael L Mumert** | **2011** | **31** | **F** | **HA,DV** | **N** | **ST** | **-** | **ND** | **ND** | **+** | **ND** |  | **GFAP** | **Yes** | **No** |
| **Maria Luisa C Policarpio-Nicolas** | **2008** | **33** | **M** | **D,HA,DV** | **ND** | **ST** | **+** | **ND** | **ND** | **+** | **ND** |  | **GFAP** | **No** | **ND** |
| **Christian Hagel** | **2009** | **34** | **F** | **ND** | **ND** | **ST** | **+** | **ND** | **ND** | **ND** | **1.0** |  | **ACTH** | **ND** | **ND** |
| **ZHU Guiwen** | **2021** | **34** | **F** | **HM,DV,VFD，Lactation** | **Co(H)** | **ST** | **+** | **+** | **ND** | **+** | **ND** | **Vimentin,Galectin-3** | **GFAP,EMA,SOX-10** | **Yes** | **ND** |
| **Abdul-kareem Ahmed** | **2019** | **36** | **F** | **N** | **PRL(H)** | **ST** | **+** | **ND** | **ND** | **ND** | **ND** | **PAS** |  | **Yes** | **Yes** |
| **Guang-Zhi Yang** | **2017** | **36** | **M** | **HA** | **Co(L)** | **ST** | **+** | **-** | **ND** | **+** | **3.0** | **TFE-3,Vimentin,CD163** | **GFAP,NSE** | **ND** | **No** |
| **Chin-Cheng Lee** | **2004** | **36** | **F** | **HM** | **PRL(H)** | **ST** | **+** | **ND** | **ND** | **ND** | **ND** | **PAS** |  | **No** | **No** |
| **Arjun V Pendharkar** | **2019** | **36** | **F** | **N** | **N** | **ST&MT** | **+** | **+** | **ND** | **ND** | **ND** | **EMA** |  | **Yes** | **No** |
| **Hai-Long Liu** | **2017** | **37** | **F** | **DV,VFD** | **N** | **ST** | **+** | **ND** | **ND** | **ND** | **1.0** | **NSE,** |  | **Yes** | **No** |
| **Akhil Mohan** | **2020** | **38** | **F** | **DV,VFD** | **Co(L)** | **ST** | **+** | **+** | **ND** | **+** | **ND** |  | **GFAP** | **Yes** | **No** |
| **Andrew Faramand** | **2018** | **40** | **F** | **HM** | **T(L),Co(L)** | **ST&MT&RT** | **+** | **ND** | **ND** | **ND** | **<1** | **PAS** | **GFAP** | **Yes** | **No** |
| **Christian Hagel** | **2004** | **41** | **M** | **ND** | **ND** | **ST** | **+** | **ND** | **ND** | **ND** | **0.0** |  | **KL1, GFAP** | **ND** | **ND** |
| **Jennifer L Orning** | **2013** | **41** | **F** | **HA,DV,VFD** | **N** | **ST&MT** | **+** | **ND** | **ND** | **ND** | **ND** | **PAS,GFAP** |  | **Yes** | **No** |
| **Akari kusakawa** | **2020** | **42** | **F** | **VFD** | **PRL(H),** | **ST** | **+** | **+** | **ND** | **ND** | **2.0** | **PAS** | **GFAP** | **Yes** | **No** |
| **G Menon** | **2008** | **42** | **M** | **DV,VFD,HA** | **GH(L),LH(L),FSH(L),Co(L)** | **ST&RT&MT** | **+** | **ND** | **ND** | **ND** | **ND** |  | **GFAP, Syn,** | **ND** | **ND** |
| **M Losa** | **2000** | **42** | **F** | **HA,Acromegaly,L** | **GH(H),IGF(H)** | **ST&MT** | **+** | **ND** | **ND** | **ND** | **ND** | **PAS,EMA,Vimentin** | **GFAP,NSE** | **Yes** | **No** |
| **Christian Hagel** | **1997** | **43** | **M** | **ND** | **ND** | **ST** | **+** | **ND** | **ND** | **ND** | **2.0** | **EMA, vimentin** | **GFAP, KL1, Syn,CgA** | **ND** | **ND** |
| **Christian Hagel** | **2010** | **43** | **M** | **ND** | **ND** | **ST** | **+** | **+** | **-** | **+** | **ND** | **GFAP, vimentin** | **KL1, Syn,CgA** | **ND** | **ND** |
| **C H Ji** | **1995** | **43** | **F** | **HA,VFD** | **ND** | **ST** | **ND** | **ND** | **ND** | **ND** | **ND** |  |  | **Yes** | **ND** |
| **Christian Hagel** | **2015** | **45** | **F** | **ND** | **ND** | **ST** | **+** | **+** | **-** | **ND** | **2.3** | **GFAP, vimentin** |  | **ND** | **ND** |
| **ＣＡＩ Ｊｉａｃｈｅｎ** | **2022** | **45** | **F** | **D** | **LH(L),FSH(H),ACTH(H)** | **ST** | **+** | **+** | **+** | **+** | **1.0** | **CD34,Syn** | **GFAP,SMA** | **No** | **No** |
| **Liu Xiaochao** | **2019** | **45** | **M** | **HA,DV** | **PRL(H)** | **ST** | **ND** | **ND** | **ND** | **ND** | **ND** | **GFAP,PAS,Syn** |  | **Yes** | **ND** |
| **Manolo Piccirilli** | **2014** | **46** | **F** | **VFD,HA** | **PRL(H),LH(L)** | **ST** | **+** | **ND** | **ND** | **ND** | **ND** | **PAS,NSE,a1-antitripsin , a1-antichy- motrypsin,** | **EMA,GFAP** | **Yes** | **No** |
| **R Bubl** | **2001** | **46** | **F** | **DV** | **N** | **ST** | **+** | **ND** | **ND** | **ND** | **ND** |  |  | **No** | **ND** |
| **Zhou Qingchen** | **2019** | **46** | **M** | **HA,DV** | **ND** | **ST** | **-** | **ND** | **ND** | **+** | **<1** | **GFAP,PAS** |  | **Yes** | **ND** |
| **Zhang Lina** | **2007** | **47** | **F** | **HA，HM** | **N** | **ST** | **+** | **ND** | **ND** | **+** | **ND** | **GFAP,NSE** | **Actin** | **No** | **No** |
| **Christian Hagel** | **2010** | **48** | **M** | **ND** | **ND** | **ST** | **+** | **ND** | **ND** | **ND** | **ND** | **GFAP, vimentin** | **KL1, Syn,CgA** | **ND** | **ND** |
| **Abdul-kareem Ahmed** | **2019** | **49** | **M** | **VFD** | **T(L),Co(L)** | **ST** | **+** | **+** | **ND** | **ND** | **ND** | **galectin-3** |  | **Yes** | **No** |
| **A Iglesias** | **2000** | **49** | **M** | **Acromegaly,L** | **ND** | **ST** | **ND** | **ND** | **ND** | **ND** | **ND** | **GFAP** |  | **Yes** | **No** |
| **A Iglesias** | **2000** | **49** | **F** | **DV,VFD** | **N** | **ST** | **ND** | **ND** | **ND** | **ND** | **ND** |  |  | **Yes** | **No** |
| **Zhu Fei** | **2000** | **49** | **F** | **HA,VFD** | **PRL(H),LH(H)** | **ST** | **+** | **ND** | **ND** | **ND** | **ND** | **PAS,A1AT,Vimentin** | **GFAP,Actin** | **Yes** | **ND** |
| **N Graziani H Dufour D Figarella-Branger P Bouillot F Grisoli** | **1995** | **50** | **M** | **HA,VFD** | **PRL(H)** | **ST** | **ND** | **ND** | **ND** | **ND** | **ND** | **PAS** |  | **No** | **No** |
| **Tu Yongbo** | **2014** | **50** | **F** | **HA,DV** | **N** | **ST** | **+** | **ND** | **ND** | **+** | **<1** |  | **Syn,GFAP,SMA** | **Yes** | **ND** |
| **Xiao Yaodong** | **2024** | **50** | **M** | **HA,VFD** | **PRL(H),E2(L),GH(L)** | **ST** | **+** | **+** | **ND** | **-** | **2.0** | **PAS,Syn,Vimentin** | **GFAP,CD163** | **Yes** | **ND** |
| **Duan Xingbang** | **2014** | **50** | **M** | **HA,D** | **N** | **ST** | **+** | **ND** | **ND** | **-** | **<1** | **NSE,CD45** | **GFAP,Syn** | **Yes** | **ND** |
| **Christian Hagel** | **2005** | **51** | **F** | **ND** | **ND** | **ST** | **+** | **+** | **-** | **+** | **ND** |  |  | **ND** | **ND** |
| **S Nishio** | **1998** | **52** | **F** | **Parkinson features** | **N** | **ST** | **+** | **ND** | **ND** | **ND** | **ND** | **GFAP** |  | **Yes** | **ND** |
| **Dou momo** | **2019** | **52** | **F** | **HA** | **PRL(H),GH(L)** | **ST** | **ND** | **ND** | **+** | **-** | **5.0** | **Vimentin,Syn** | **GFAP,SOX-10,PAS** | **Yes** | **ND** |
| **Franco Rubino** | **2020** | **53** | **F** | **HA,VFD** | **N** | **ST** | **ND** | **+** | **ND** | **+** | **4.2** |  |  | **ND** | **No** |
| **K Aquilina** | **2006** | **53** | **M** | **HA,DV,VFD,L** | **PRL(H)** | **ST** | **ND** | **ND** | **ND** | **+** | **ND** | **PAS,GAFP,NSE,A1AT** |  | **Yes** | **No** |
| **Haitao Wu** | **2024** | **55** | **F** | **N** | **ND** | **ST** | **-** | **ND** | **-** | **+** | **1.0** | **Vimentin** | **GFAP** | **Yes** | **ND** |
| **Takashi Shizukuishi** | **2014** | **55** | **M** | **N** | **N** | **ST** | **+** | **ND** | **ND** | **ND** | **1.0** | **PAS,NSE** |  | **Yes** | **No** |
| **T K Kobayashi** | **2006** | **55** | **F** | **DV,VFD** | **ND** | **ST** | **+** | **ND** | **ND** | **ND** | **ND** | **PAS,GFAP** |  | **No** | **No** |
| **Wang. Mingliang** | **2021** | **55** | **F** | **D** | **N** | **ST** | **+** | **ND** | **+** | **ND** | **3.0** | **PAS,Vimentin,NSE,GFAP,Syn** |  | **No** | **ND** |
| **Gianluca Lopez** | **2023** | **56** | **M** | **VFD,DV,HA** | **ND** | **ST** | **+** | **+** |  | **+** | **ND** | **Vimentin,GFAP,CD31,Annexin-A** | **EMA,OLIG2,CD34,CD163,WT1,p53 , PAX8** | **No** | **ND** |
| **Dario Gagliano** | **2024** | **56** | **M** | **HA,VFD** | **ND** | **ND** | **ND** | **ND** | **ND** | **ND** | **ND** |  |  | **Yes** | **ND** |
| **Li Bowei** | **2024** | **56** | **F** | **HA,DV** | **PRL(H)** | **ST** | **+** | **+** | **ND** | **+** | **8.0** | **Vimentin,GFAP** | **Syn** | **Yes** | **No** |
| **Christian Hagel** | **2012** | **57** | **F** | **ND** | **ND** | **ST** | **+** | **ND** | **ND** | **+** | **ND** | **GFAP, NSE MAP2** |  | **ND** | **ND** |
| **Franco Rubino** | **2020** | **57** | **M** | **VFD** | **ND** | **ST** | **ND** | **+** | **ND** | **ND** | **2.0** |  |  | **ND** | **No** |
| **Wang Shan** | **2016** | **57** | **F** | **DV** | **ND** | **ST** | **+** | **ND** | **+** | **-** | **5.0** | **PAS,EMA,Syn,Vimentin** | **GFAP,CEA** | **Yes** | **ND** |
| **Fernando Guerrero-Pérez** | **2019** | **58** | **F** | **VL** | **ND** | **ST** | **-** | **+** | **-** | **ND** | **3.0** | **Vimentin,** | **GFAP** | **ND** | **ND** |
| **D H Becker** | **1981** | **58** | **M** | **VFD** | **ND** | **ST&RT** | **ND** | **ND** | **ND** | **ND** | **ND** | **PAS,GFAP** |  | **ND** | **ND** |
| **Abdul-kareem Ahmed** | **2019** | **60** | **M** | **HA** | **ND** | **ST&MT** | **+** | **ND** | **ND** | **ND** | **ND** | **PAS,GFAP** |  | **Yes** | **No** |
| **P Saint-Blancard** | **2007** | **60** | **M** | **HA,VFD** | **T(L),TESTO(L)** | **ST** | **+** | **ND** | **ND** | **ND** | **<2%** | **PAS,NSE** | **GFAP** | **Yes** | **ND** |
| **Christian Hagel** | **2008** | **61** | **M** | **ND** | **ND** | **ST** | **+** | **+** | **-** | **+** | **ND** |  |  | **ND** | **ND** |
| **Christian Hagel** | **2006** | **62** | **F** | **ND** | **ND** | **ST** | **+** | **+** | **-** | **+** | **ND** | **GFAP, vimentin** | **KL1, CgA** | **ND** | **ND** |
| **H Kawano** | **1989** | **62** | **M** | **VFD,VL** | **ND** | **ST&RT** | **ND** | **ND** | **ND** | **ND** | **ND** | **GFAP** |  | **Yes** | **Yes** |
| **D J Halbauer** | **2003** | **63** | **M** | **VFD** | **ND** | **ST** | **+** | **ND** | **ND** | **ND** | **ND** | **PAS,NSE,KP-1** | **GFAP,Vimentin** | **No** | **No** |
| **S Kasashima** | **2000** | **63** | **F** | **VFD** | **ACTH(H),T3(L),T4(L),FSH(L),LH(L)** | **ST&RT** | **+** | **ND** | **ND** | **ND** | **7.0** | **PAS,NSE,Vimentin, P53** | **GFAP,** | **Yes** | **No** |
| **Zhang Haifeng** | **2018** | **63** | **M** | **N** | **T3(H) PRL(H)** | **ST** | **+** | **+** | **ND** | **-** | **<2** | **CD3** | **GFAP,NSE,PAS, CD38** | **Yes** | **ND** |
| **Ujjawal Khurana** | **2020** | **65** | **F** | **VFD** | **ND** | **ST** | **+** | **+** | **ND** | **+** | **2.0** | **Vimentin,GFAP,EMA** | **Pancytokeratin, Syn** | **Yes** | **ND** |
| **John S Rhee** | **2002** | **65** | **M** | **D,HA** | **N** | **ST** | **+** | **ND** | **ND** | **ND** | **ND** | **PAS** | **EMA,AE-I,KP-I** | **No** | **No** |
| **Hai-Long Liu** | **2017** | **66** | **F** | **D** | **N** | **ST** | **+** | **ND** | **ND** | **+** | **1.0** | **GFAP,** |  | **No** | **No** |
| **Robert C Rennert** | **2022** | **66** | **F** | **HA** | **N** | **ST** | **ND** | **ND** | **ND** | **ND** | **ND** |  |  | **No** | **No** |
| **Fernando Guerrero-Pérez** | **2019** | **66** | **M** | **HA** | **ND** | **ST** | **+** | **+** | **+** | **ND** | **1～2** | **GFAP** |  | **ND** | **ND** |
| **Christian Hagel** | **1993** | **68** | **F** | **ND** | **ND** | **ST** | **+** | **ND** | **ND** | **ND** | **ND** | **Vmientin** | **EMA,KL1, GFAP,CEA** | **ND** | **ND** |
| **A Gregoire** | **2015** | **68** | **M** | **VFD** | **ND** | **ST** | **ND** | **ND** | **ND** | **ND** | **ND** | **PAS** |  | **Yes** | **No** |
| **Yi Zhang** | **2018** | **69** | **F** | **DV,VFD,HA,D** | **PRL(H),E2(L),LH(L),FSH(L),Co(L),TSH(L)** | **ST** | **+** | **ND** | **-** | **ND** | **6.0** | **PAS,Syn** |  | **Yes** | **No** |
| **Shun Yamamuro** | **2017** | **70** | **F** | **VFD** | **N** | **ST** | **+** | **+** | **ND** | **+** | **ND** | **EMA,** | **GFAP,Syn,CgA** | **Yes** | **ND** |
| **Yufei Dai** | **2019** | **70** | **F** | **DV,VFD,HA** | **N** | **ST** | **+** | **+** | **ND** | **ND** | **1.0** | **PAS** | **Syn** | **Yes** | **No** |
| **Christian Hagel** | **2007** | **72** | **F** | **ND** | **ND** | **ST** | **+** | **ND** | **ND** | **ND** | **1.0** |  | **P53** | **ND** | **ND** |
| **B Schaller** | **1998** | **75** | **F** | **HD,VFD,D** | **FSH(L),IGF-1(L)** | **ST&RT** | **-** | **ND** | **ND** | **ND** | **ND** | **PAS** | **Vmientin** | **Yes** | **No** |
| **E Moriyama** | **1996** | **75** | **F** | **N** | **N** | **ST** | **+** | **ND** | **ND** | **ND** | **ND** | **PAS** | **GFAP** | **No** | **No** |
| **R Bubl** | **2001** | **76** | **F** | **DV,VFD** | **ND** | **ST** | **+** | **ND** | **ND** | **ND** | **ND** |  | **GFAP** | **Yes** | **ND** |
| **Christ0pher S Hong** | **2021** | **77** | **F** | **VFD** | **ND** | **ST&RT** | **+** | **+** | **ND** | **+** | **2.0** | **PAS** | **GFAP, Syn,PAX8, D2-40** | **Yes** | **No** |
| **D H Becker** | **1981** | **78** | **F** | **DV,VFD** | **N** | **ST&RT** | **ND** | **ND** | **ND** | **ND** | **ND** | **PAS** |  | **No** | **ND** |
| **Christian Hagel** | **2009** | **86** | **M** | **ND** | **ND** | **ST** | **+** | **+** | **-** | **+** | **6.0** |  |  | **ND** | **ND** |

F=female; M=male; D=dead; A=alive; Mo=month; ND=not described; N=normal;DV= decreased vision ; VL=vision loss; VFD=visual field defect; HA=headache; DI=diabetes insipidus; LH(L)=low luteinizing hormone;P=Polyuria;TSH(L)=low thyroid-stimulating hormone; FSH(L)=low follicle-stimulating hormone; PRL(H)=high prolactin; Co(L)=low cortisol; ACTH(L)=low adreno-cortico-tropic hormone; T(L)=low thyroxine; T(H)=high thyroxine R=rich; RT=radiation therapy; ST=surgical treatment; MT=medical treatment; HM=amenorrheic; D=Dizziness; LA= Lactation; L= lethargy;
